# A randomized controlled trial comparing rehabilitation with isokinetic exercises and Thera-Band strength training in patients with functional ankle instability

**DOI:** 10.1371/journal.pone.0278284

**Published:** 2022-12-01

**Authors:** Bin Wang, Xi Zhang, Feilong Zhu, Weiwei Zhu, Xinyu Wang, Fan Jia, Wei Chen, Ming Zhang

**Affiliations:** 1 The Affiliated Xuzhou Rehabilitation Hospital of Xuzhou Medical University, Xuzhou Rehabilitation Hospital, Xuzhou, China; 2 Department of Rehabilitation Medicine, Xuzhou Central Hospital, The Xuzhou Clinical College of Xuzhou Medical University, Xuzhou, China; 3 Beijing Sport University, Beijing, China; 4 College of Physical Education and Sports, Beijing Normal University, Beijing, China; Public Library of Science, UNITED STATES

## Abstract

**Background:**

Although muscle strength training is a prevalent treatment for patients with functional ankle instability (FAI), previous investigations on the efficacy have yielded conflicting results.

**Objective:**

This study aims to compare the efficacy of 6-week isokinetic strength training and Thera-Band strength training on improving ankle strength, dynamic balance ability, and function in individuals with FAI.

**Methods:**

Fifty-two FAI patients were randomized into two treatment groups: an isokinetic strength training (IST, n = 26) group and a Thera-Band resistance training (TBT, n = 26) group. The IST group engaged in isokinetic concentric strength training with inversion, eversion, dorsiflexion, and plantar flexion, whereas the TBT group engaged in progressive resistance training with Thera-Band three times per week for six weeks. Before and after the training, an isokinetic concentric strength test of the involved ankle joint, Star Excursion Balance Test (SEBT), and Cumberland Ankle Instability Tool (CAIT) function assessment were performed.

**Results:**

After six weeks of intervention, the strength of inversion and eversion was significantly improved in both the IST and TBT groups (*p* < 0.05), with the IST group exhibiting a significant (*p* < 0.05) improvement when compared to the TBT group. The SEBT and CAIT results were significantly (*p* < 0.05) improved in the IST group compared to the TBT group.

**Conclusions:**

The six-week of isokinetic strength training is more effective than the Thera-Band progressive resistance training in improving the physical function of FAI patients.

**Clinical trial registration number:**

This randomized controlled clinical trial has been registered in the China Clinical Trial Registry (ChiCTR2100044444) https://www.google.com/search?client=firefox-b-d&q=ChiCTR2100044444.

## Introduction

Acute ankle sprain is one of the most prevalent joint injuries among human musculoskeletal injuries, typically occurring during daily activities and many sports [1]. Most of ankle joint sprains occur when the ankle joint is in an inverted and plantar flexed position, typically resulting in an ankle lateral ligament injury [2]. The literature indicates that approximately 40% of ankle injuries are accompanied with persistent ankle instability and weakness. These residual symptoms were described as the sensation of a “giving way” by individuals who self-reported ankle instability, which was defined as functional ankle instability (FAI) [3]. Subsequent studies have reported the clinical manifestations of FAI, in which motion is physiological but no longer under voluntary control [4]. Though insufficient muscle strength [5–8], proprioceptive dysfunction [7,9–11], postural control disorders [12–15], and neuromuscular control disorders [16–18] have been confirmed by several studies to be present in the FAI population, the pathogenesis of functional ankle instability has not been comprehensively elucidated. Due to the injury of the mechanical receptors of involved joint caused by the first ankle sprain, the signal transduction process failed, resulting in the muscle force imbalance of the involved ankle joint caused by repeated ankle joint sprains [17].

Numerous investigations have confirmed the deficiency of the FAI peri-ankle strength since the development of isokinetic muscle strength testing techniques [19,20]. Therefore, the majority of the clinical interventions include isokinetic muscle strength training or progressive strength programs. However, the muscle strength, balance, and functional training effects of the FAI population are contradictory due to the inconsistency of training programs [21–24]. To determine which peri-ankle muscle strength training can improve function in FAI patients, further investigations are required on the isokinetic muscle strength training and Thera-Band strength training. Sekir et al. [23] observed improvements in the peri-ankle strength, joint position sense, and ankle function in FAI athletes with the isokinetic muscle strength training compared to pre-training. Kaminski et al. [22] observed no significant improvement in the strength of the evertor and invertor muscles after the Thera-Band progressive resistance training.

No studies on more effective and less costly training methods for FAI patients have been reported yet. Due to the complex mechanisms of injury in patients with FAI, a clinical intervention cannot be evaluated solely based on improvements in individual indicators, such as muscle strength. The capacity of the training program allows the patient to restore normal physical activity should be the primary concern. Although isometric strength training devices are available in basically every hospital in China, The Thera-Band progressive resistance strength training is used to treat the majority of FAI patients. It is not clear whether there is a difference in the efficacy between the isometric plyometric training and Thera-Band progressive resistance training in improving patients with FAI.

The present study is a preliminary exploration of a more cost-effective and practicable training protocol for FAI patients. To this aim, a randomized controlled trial was performed to compare the efficacy between the isokinetic concentric strength training and Thera-Band strength training for six weeks on ankle strength, dynamic balance ability, and function in the FAI populations. The results exhibit that six weeks of isokinetic concentric strength training is more effective than the Thera-Band progressive resistance training for functional improvements in FAI patients.

## Materials and methods

### 2.1 Study design and subjects

This randomized controlled trial has been registered in the China Clinical Trial Registry (ChiCTR2100044444). Data collection was performed at Xuzhou Rehabilitation Hospital, Xuzhou Medical University. Fifty-two subjects were randomized into two treatment groups using a random number table: an isokinetic muscle training (IST, n = 26) group and a Thera-Band Training (TBT, n = 26) group. Each ID number corresponded to a sealed envelope with the group assignment. An informant who was not involved in the outcomes assessments completed this procedure. An experienced physiotherapist who was blinded to group assignment and did not participate in the intervention performed all assessments of outcomes. The study process was approved by the review board of Xuzhou Rehabilitation Hospital (ethical approval code: XKYL2021004). All subjects were informed of the purpose of the study and related experimental procedures and signed an informed consent form.

**Fig 1:**
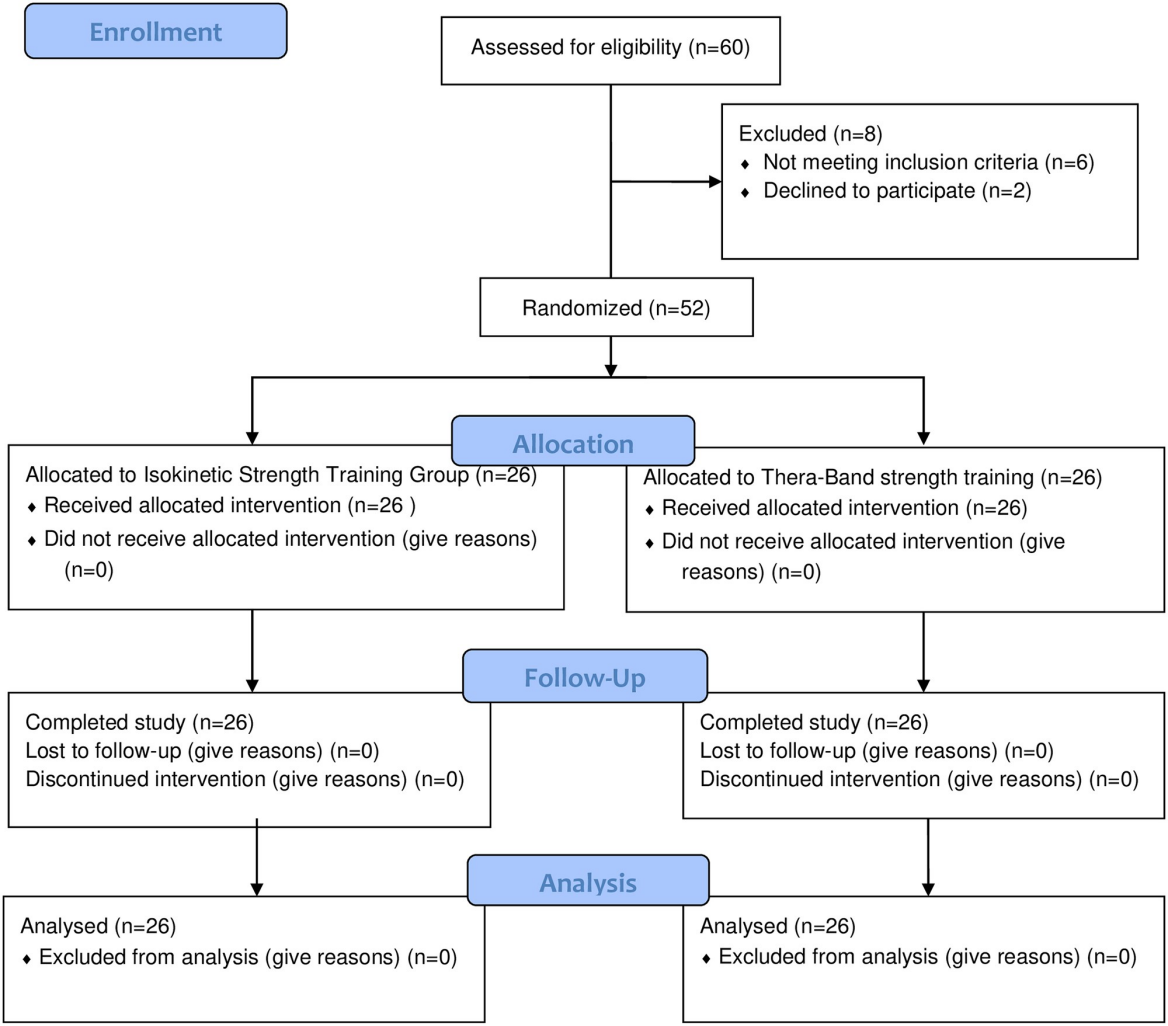
Consolidated standards of reporting trials flow chart.

According to the inclusion and exclusion criteria, 52 individuals with unilateral FAI were included, all of them met the diagnostic criteria for chronic ankle instability established by the International Ankle Consortium [25]. From April 2021 to November 2021, the individuals were recruited from the Xuzhou Central Hospital, the Affiliated Xuzhou Rehabilitation Hospital of Xuzhou Medical University, and online platforms (WeChat, QQ). A CONSORT diagram is illustrated in Fig 1.

The following were the inclusion criteria: (1) Patients over the age of 18 years; (2) unilateral ankle sprain, anterior drawer and talar tilt test by the same clinician did not discover significant structural instability; (3) unilateral ankle joint had at least one significant history of lateral ankle sprain in the past 1 year, swelling or pain after an injury resulting in the inability of the involved foot to bear weight normally, and loss of ankle joint control during functional activities; (4) an acute ankle sprain occurred more than 3 months before the enrollment; (5) no other severe injuries in the lower limbs and ankle joints, such as the fractures, surgery, etc.; (6) the Cumberland Ankle Instability Tool (CAIT) score was < 24 points [26]; (7) the injured ankle had not received rehabilitation treatments; (8) voluntarily participated in this study and signed an informed consent form. Exclusion criteria: (1) Bilateral ankle sprains; (2) the history of fracture or surgery in lower limbs; (3) ankle joint talar tilt test and anterior drawer test were positive, excluding mechanical ankle instability; (4) suffering from other neurological diseases affecting muscle strength and balance.

### 2.2 Procedures

Before the training, all individuals underwent the CAIT score, isokinetic muscle strength, and dynamic balance tests. The IST group and TBT group all received prescribed muscle strength exercise programs consisting of 3 days per week for 6 weeks. All the tests and trainings were completed in the Sports Medicine Department of the Affiliated Xuzhou Rehabilitation Hospital of Xuzhou Medical University. All individuals were reassessed after six weeks using the following outcome measures: isokinetic muscle strength and dynamic balance.

#### 2.2.1 Isokinetic muscle strength test

A multi-joint isokinetic muscle strength testing and training system (Guangzhou Yikang Medical Equipment Industrial Co., Ltd. A8-2 type, China) device was used in this study. Before the test, the individual was positioned supine on the seat. The equipment was adjusted and secured in strict accordance with the equipment safety manual, taking into account height, body size, *etc*. First, under the condition of 60°/s angular velocities, each individual was allowed to perform three maximal concentric exercises of ankle dorsiflexion and plantarflexion. Then individuals were randomized into the IST and TBT groups for the isokinetic muscle strength test after 30 minutes of rest to avoid the effects of learning and fatigue. Under the conditions of 60°/s and 120°/s angular velocities, each individual completed 10 consecutive repeated maximum isokinetic concentric contractions of ankle dorsiflexion and plantarflexion, inversion and eversion, and the peak torque and peak torque/body weight were recorded. The selected evaluation indicators included relative peak torque (RPT) and the ratio of peak torque to individual body mass. In this study, the effect of individual body mass on muscle strength was minimized, allowing us to compare the differences in muscle strength caused by weight differences [27].

#### 2.2.2 Balance test

The Star Excursion Balance Test (SEBT) was used for the balance test because of its sufficient sensitivity and high retest reliability in the ankle dynamic balance test [28,29]. The exact test procedures were as follows: firstly, leg length was measured from the anterior superior iliac spine to the medial malleolus on physical examination by the same therapist; secondly, each individual was asked to stand barefoot with the navicular of their stance limb positioned over the center of the SEBT tape grid, both of their hands placed on the waist and bearing weight on the unilateral lower limb to maintain physical stability. In order to minimize the effects of learning and order, the formal test was performed in either a clockwise or a counter-clockwise direction (when the affected leg was the right leg, we used a counter-clockwise order for the test; when the patient’s affected leg was the left leg, we used a clockwise direction). All individuals were tested in the following order: anterior, anterolateral, lateral, posterolateral, posterior, posteromedial, medial, and anteromedial. The maximal distance that the foot was lightly touched in each direction was recorded, with greater distance indicating greater ankle joint stability. The ratio of maximal extension distance to leg length was calculated as an index of dynamic balance capability.

#### 2.2.3 CAIT scoring

The Cumberland Ankle Instability Tool (CAIT), consisting of nine questions with high reliability and validity, was used to measure FAI [30,31]. Before the trial, the CAIT was used to assess the severity of FAI in individuals. After the end of training, the individuals were rated again to assess their recovery status. Before distributing the questionnaires, the researchers introduced themselves and explained the plan and the questions to the individuals.

### 2.3 Strength protocols

**Fig 2:**
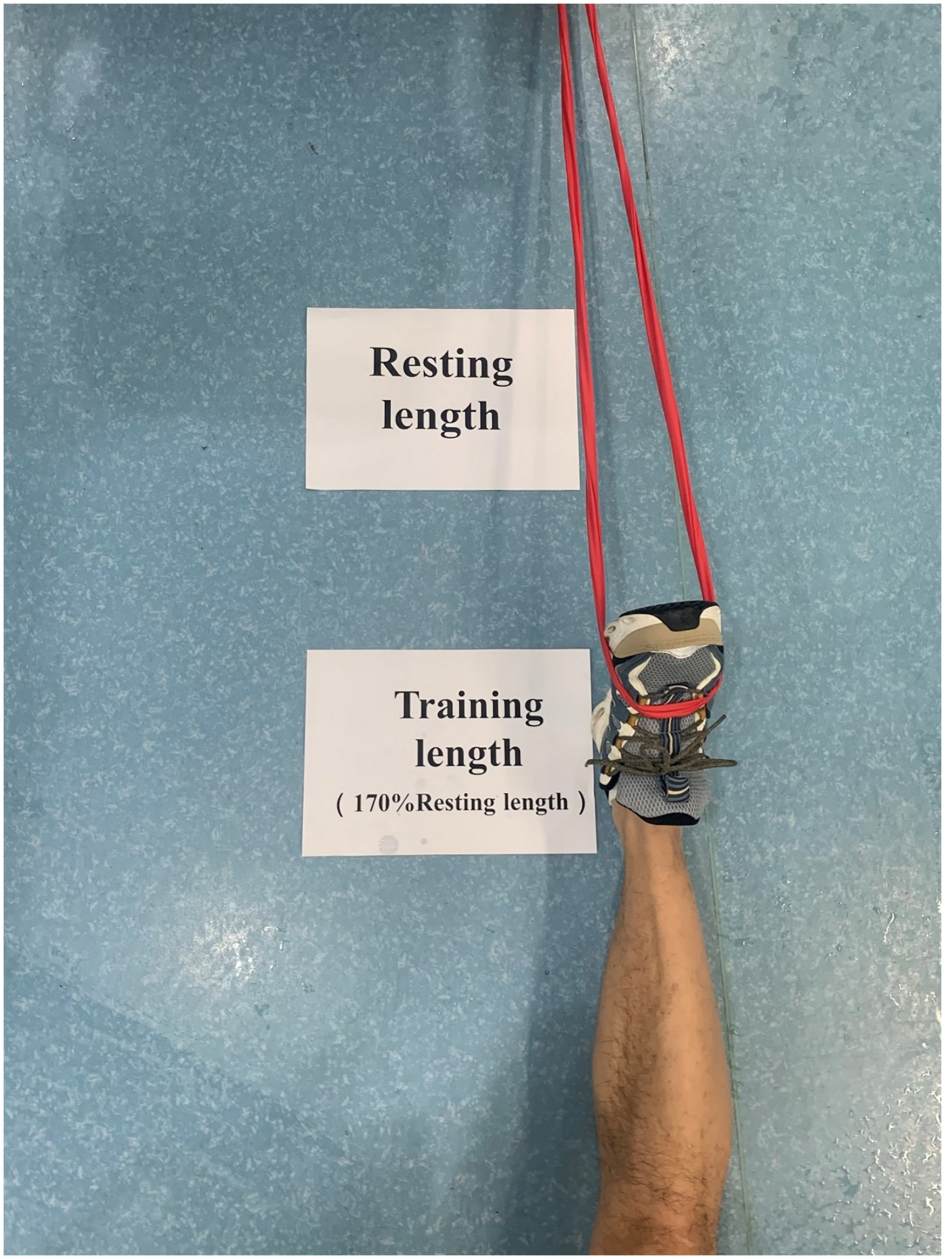
Thera-band resistance training.

**Fig 3:**
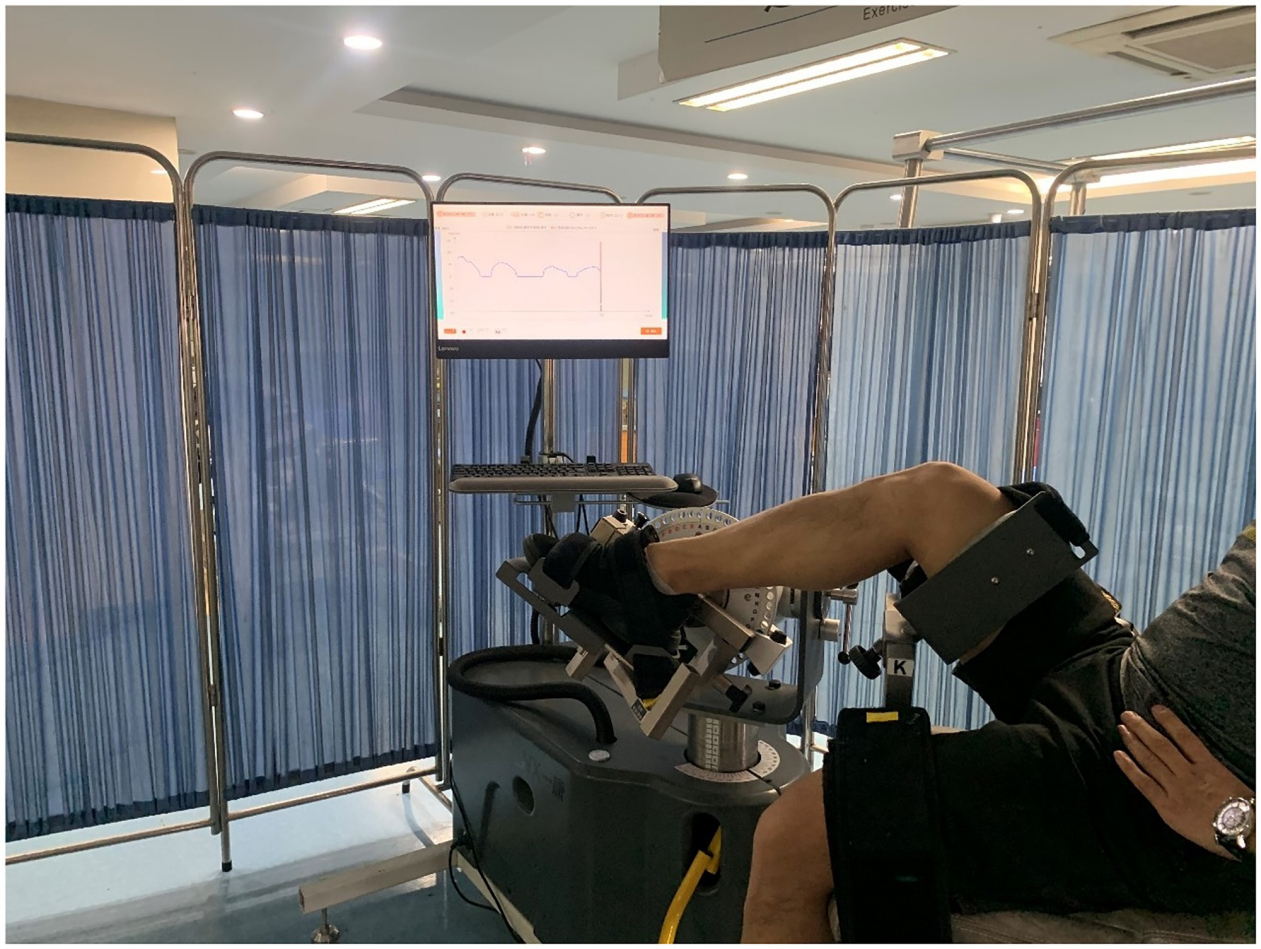
Isokinetic strength training.

The TBT group performed the Thera-Band progressive resistance training. During the TBT resistance training, individuals received progressive resistance concentric contractions of dorsiflexion, plantarflexion, invertor and evertor muscles, and only the ankle perimeter muscle was trained without compensations of the knee and hip joints (Fig 2). The 170% resting length was selected as the resistance starting point in the Thera-Band to ensure greater resistance and standardization [32]. To match the corresponding muscle contraction pattern, the IST group performed the isokinetic concentric contractions strength training, with primary dorsiflexion, plantarflexion muscle strength exercises, and a subsequent ankle invertor and evertor isokinetic muscle strength training after 5 minutes of rest. The concentric contractions were used as training procedures at an angular velocity of 60°/s, according to the A8-2 training manual. The dorsiflexion and plantarflexion muscle strengths were trained in a dorsiflexion/plantarflexion movement pattern, whilst the ankle invertor and evertor muscle strengths were trained in an inversion/eversion movement pattern (Fig 3).

Treatments duration and intensity were the same for both groups throughout the whole six-week exercise cycle: 40 minutes, three times per week. Furthermore, the individuals were prohibited from engaging in any activities except the training program during the study period.

### 2.4 Data analysis

Statistical analysis was performed using IBM SPSS Statistics 25 software (SPSS Inc, Chicago, Illinois). The level of significance was set at *P* < 0.05. The normality of each variable was initially tested using the Kolmogorov-Smirnov test. The chi-square test was performed to compare values for difference of gender. The independent sample t-test was performed to compare data from two groups, while the paired t-test was performed to compare data from individuals within the same group. The effect sizes after training for the two interventions were calculated using Cohen’s d. The sample size was estimated with the G*Power 3.1.9.2 software based on some similar studies [5,19]. Consequently, based on a statistical power of 0.8, alpha = 0.05, and an estimated effect size = 0.7, the estimated sample size was determined to be 26 individuals for each group (total = 52).

## Results

From April 2021 to November 2021, individuals were recruited from the Xuzhou Rehabilitation Hospital Affiliated to Xuzhou Medical University (Xuzhou Central Hospital) and network platforms (WeChat, QQ). Sixty individuals participated in the recruitment. Some of the participants did not meet the inclusion criteria (n = 6), while others refused to participate (n = 2). Finally, 52 individuals were eligible.

### 3.1 Characteristics of the included individuals

Table 1 shows the demographic features of the included individuals with no significant differences in these variables.

**Table 1:** Characteristics of the IST and TBT groups.

Characteristics	IST group (n = 26)	TBT group (n = 26)	*P* value
Age (years)	39.92 (9.92)	38.81 (9.44)	0.680
Gender (woman/man)	14/12	11/15	0.782
Height (cm)	166.04 (9.53)	168.38 (9.51)	0.378
Weight (kg)	67.19 (9.47)	68.96 (7.89)	0.468
BMI (kg/m^2^)	24.58 (4.54)	24.46 (3.28)	0.908

### 3.2 Strength analysis

Table 2 shows the results of isokinetic muscle strength tests at 60°/s and 120°/s after six weeks of treatments. Invertor and evertor muscle strengths were improved after training in both the IST and TBT groups. A statistically significant (*p* <0.001) difference in invertor and evertor muscle strengths was observed between the two groups. Conversely, no significant difference in dorsiflexion and plantar-flexion muscle strengths was observed between the two groups.

**Table 2:** Muscle strength of the ankle at 60 and 120 degrees/sec angular velocities in the IST and TBT groups before and after the interventions.

Group		IST group	TBT groups	*P* value	Effect size
Inv RPT (Nm/Kg)	60°/s Pre-training	0.24 (0.05)	0.22 (0.05)	0.305	
	60°/s Post-training	0.39 (0.09)	0.30 (0.05)	0.001	0.526
	*P* value	0.001	0.001		
	120°/s Pre-training	0.19 (0.04)	0.18 (0.03)	0.101	
	120°/s Post-training	0.30 (0.06)	0.25 (0.06)	0.001	0.385
	*P* value	0.001	0.001		
Eve RPT (Nm/Kg)	60°/s Pre-training	0.19 (0.04)	0.17 (0.04)	0.189	
	60°/s Post-training	0.33 (0.07)	0.25 (0.07)	0.001	0.496
	*P* value	0.001	0.001		
	120°/s Pre-training	0.15 (0.03)	0.13 (0.04)	0.206	
	120°/s Post-training	0.25 (0.06)	0.17 (0.04)	0.001	0.617
	*P* value	0.001	0.003		
Dorsi RPT (Nm/Kg)	60°/s Pre-training	0.37 (0.07)	0.37 (0.07)	0.968	
	60°/s Post-training	0.37 (0.09)	0.39 (0.06)	0.514	0.130
	*P* value	0.976	0.465		
	120°/s Pre-training	0.23 (0.06)	0.23 (0.04)	0.954	
	120°/s Post-training	0.25 (0.07)	0.24 (0.04)	0.700	0.087
	*P* value	0.129	0.131		
Plantar RPT (Nm/Kg)	60°/s Pre-training	0.44 (0.08)	0.45 (0.07)	0.564	
	60°/s Post-training	0.44 (0.08)	0.46 (0.10)	0.988	0.110
	*P* value	0.219	0.882		
	120°/s Pre-training	0.32 (0.06)	0.29 (0.05)	0.066	
	120°/s Post-training	0.32 (0.06)	0.31 (0.05)	0.757	0.090
	*P* value	0.360	0.352		

Inv indicates Inversion; Eve indicates Eversion; Dorsi indicates Dorsiflexion; Plantar indicates Plantar flexion; RPT, relative peak torque.

### 3.3 Dynamic balance analysis

Table 3 exhibits the dynamic balance of the ankle before and after the 6-week strength interventions. The balance ability was significantly improved in the eight directions of the IST group, whereas no significant difference was observed in any direction of the TBT group. Specifically, the IST treatment improved the balance ability after training, whereas the TBT treatment did not after training.

**Table 3:** Balance ability of the IST and TBT groups revealed by the SEBT score before and after the interventions.

Group		IST group	TBT group	*P* value	Effect size
Anterior	Pre-training	0.63 (0.12)	0.62 (0.07)	0.755	
	Post-training	0.82 (0.08)	0.62 (0.06)	0.001	0.816
	*P* value	0.001	0.689		
Anterolateral	Pre-training	0.73 (0.08)	0.70 (0.09)	0.184	
	Post-training	0.82 (0.08)	0.69 (0.08)	0.001	0.631
	*P value*	0.001	0.536		
Lateral	Pre-training	0.73 (0.10)	0.70 (0.09)	0.358	
	Post-training	0.88 (0.09)	0.72 (0.06)	0.001	0.723
	*P value*	0.001	0.351		
Posterolateral	Pre-training	0.62 (0.07)	0.58 (0.08)	0.109	
	Post-training	0.74 (0.11)	0.61 (0.06)	0.001	0.592
	*P value*	0.001	0.194		
Posterior	Pre-training	0.66 (0.08)	0.64 (0.07)		
	Post-training	0.83 (0.07)	0.63 (0.09)	0.001	0.778
	*P value*	0.001	0.422		
Posteromedial	Pre-training	0.63 (0.06)	0.59 (0.10)	0.125	
	Post-training	0.70 (0.07)	0.60 (0.06)	0.001	0.609
	*P value*	0.001	0.834		
Medial	Pre-training	0.42 (0.08)	0.39 (0.07)	0.121	
	Post-training	0.54 (0.07)	0.41 (0.05)	0.001	0.730
	*P value*	0.001	0.212		
Anteromedial	Pre-training	0.46 (0.08)	0.42 (0.07)	0.089	
	Post-training	0.62 (0.07)	0.42 (0.07)	0.001	0.819
	*P value*	0.001	0.529		

### 3.4 CAIT scoring analysis

Table 4 shows the CAIT scores of the IST and TBT groups before and after six weeks of isokinetic muscle strength training and Thera-Band muscle strength resistance training. The IST treatment resulted a significant difference in CAIT scores before and after training, however, the TBT treatment led to an increase in the CAIT score, albeit the difference was not statistically significant.

**Table 4:** Scores of CAIT in the IST and TBT groups.

Group	IST group	TBT group	*P* value	Effect size
Before	17.46 (2.96)	17.62 (3.51)	0.865	
After	26.38 (1.86)	18.15 (4.07)	0.001	0.793
*P value*	0.001	0.427		

## Discussion

FAI is characterized by core symptoms of insufficient muscle strength, proprioceptive dysfunction, and postural and neuromuscular control disorders. The current randomized controlled trial was performed to compare the efficacy between the isokinetic concentric strength training and Thera-Band strength training for six weeks on ankle strength, dynamic balance ability, and function in the FAI populations. As expected, the current study shows that the 6-week isokinetic concentric strength training is more effective than the Thera-Band progressive resistance training for functional improvements in FAI patients.

During daily activities, various muscle groups surrounding the ankle joint may fire individually to flex or extend the joint and maintain physical stability. Insufficient ankle evertor muscle strength reduces the ability to resist varus when ankle sprains occur and the ankle joint cannot return to its neutral position promptly, resulting in repetitive ankle sprains. In recent years, both isokinetic muscle strength training and Thera-Band strength training have been used extensively to increase muscle strength after sports injuries and improve muscle performance in athletes, thereby accelerating injury recovery [33–35]. The current study aims to compare between the efficacy of 6 weeks of isokinetic training and Thera-Band strength training on muscle strength, balance ability, and self-reported functional scores in the ankle joint. The current study exhibits the remarkable improvement of ankle strength after 6 weeks of muscle strength training. IST treatment significantly (*p* < 0.05) improved the eversion and inversion of concentric strength at test angular velocities of 60°/s and 120°/s, corroborating previous findings [21,23]. Uh et al. [23] performed eight weeks of isokinetic muscle strength training with eccentric and concentric motion modes. The results of the study revealed that the ankle inversion and eversion muscle strengths were significantly improved after training when compared to the pre-training values. Similarly, TBT treatment significantly improved evertor and invertor muscle strengths, which is consistent with previous findings [24,36].

However, current findings are contradicted by other studies. For example, Kaminski *et al*. [22] demonstrated that a six-week Thera-Band strength training program did not improve muscle strength of the involved ankle joint in FAI patients. The reason for the discrepancies might be due to that the eccentric muscle strength test was performed by Kaminski *et al*., while the concentric strength test was performed in the current study. Surprisingly, no improvements in ankle dorsiflexion and plantarflexion strengths were observed in both the IST and TBT groups. One possible explanation is that the dorsiflexor and plantar-flexor muscle groups are frequently used during walking and daily activities. Thus, our strength training yielded no improvements in these two muscle groups. More importantly, the current study exhibits that the IST treatment may have a greater effect than the TBT treatment. The difference was observed based on the muscle contraction pattern adopted during training between the two groups. The concentric contraction was used in both groups. Therefore, the current study cannot determine whether this difference is caused by the isokinetic contraction of muscle fibers during the isokinetic muscle strength training mode or whether the isokinetic muscle strength training provides greater resistance than Thera-Band.

Ankle instability can impair standing balance and even the stability of movements such as walking and jumping. The current study exhibits that IST treatment can significantly improve the balance ability in all directions. This improvement was mainly attributed to that the compliance resistance provided by isokinetic training could better stimulate the mechanical receptors surrounding the ankle, hence helping FAI patients perform well in balance function tests. Conversely, no statistically significant difference was found in dynamic ability in the TBT group. Balance ability is inseparable from proprioception. Therefore, it is possible that TBT treatment does not cause excitation of peri-ankle mechanical receptors and does not play a corresponding role in the recovery of proprioception. The absence of proprioception assessment is a limitation in the current study. Smith et al. [24] observed that 6-week TBT treatment improves peri-ankle strength but not proprioception after interventions. This is a possible explanation for no improvement of the balance ability in the TBT group in the current study. Similarly, Powers et al. [37] observed the improvement of fatigue and balance ability of FAI patients with proprioception combined with muscle strength training for 6 weeks, whereas muscle strength training alone, proprioception training, and their combination training could not improve the static balance ability. According to aforementioned studies, all training cycles are three days per week for six weeks. It may be possible to conclude that the lack of 6-week Thera-Band strength training protocol produces no significant changes. Thus, a training cycle greater than 6 weeks or training more frequently, or a combination of the two, can perhaps improve balance ability.

However, an optimal training program should not only focus on muscle strength and balance ability but also on whether the training protocol improves functions. The CAIT is an indicator to assess and diagnose FAI functions. Our study exhibited that IST treatment for six weeks significantly (*p* < xxx) improved self-reported functional scores in FAI individuals, but the TBT treatment showed no improvement in self-reported functional scores. The following reasons could be the possible explanations: I) The imbalance of evertor and invertor muscle strengths may lead to a biomechanical imbalance of the ankle joint; II) individuals feel that the ankle joint lost control during daily activities, and III) repetitive sprains. After isokinetic concentric strength training, the balance of ankle joint muscle strength can restore better. Similarly, Sekir et al. [23], in a 6-week isokinetic concentric strength training, demonstrated that the functional capacity of the injured ankle reached the same level as the healthy ankle. This indicates that the isokinetic muscle strength training can improve functional performance in FAI patients. The aforementioned results were not observed in the TBT group, possibly because the resistance provided by the Thera-Band force during specific training was not sufficient to stimulate the receptors surrounding the ankle joint, and the patient could not establish good functional feedback to improve the self-reported scores.

### Limitations

A proprioception test after IST or TBT training is absent in the current study. Thus, whether the functional changes in the IST group are due to the enhancement of the proprioception of FAI individuals by isokinetic muscle training is not clear. Further investigation is required to focus on whether the proprioception of FAI individuals changes before and after isokinetic muscle training.

## Conclusion

To conclude, the six-week isokinetic concentric strength training not only improves the strength and balance ability to restore ankle inversion/eversion muscles but also exhibits a more significant advantage in restoring functions than the Thera-Band strength training in FAI patients.

## Supporting information

S1 File CONSORT checklist. (DOC)

S2 File (DOCX)

S3 File (PDF)

## References

[1] Vuurberg G, Hoorntje A, Wink LM, van der Doelen BFW, van den Bekerom MP, Dekker R et al. Diagnosis, treatment and prevention of ankle sprains: update of an evidence-based clinical guideline. Br J Sports Med 2018, 52(15):956. doi: 10.1136/bjsports-2017-098106 . 29514819

[2] Hertel J. Functional Anatomy, Pathomechanics, and Pathophysiology of Lateral Ankle Instability. J Athl Train 2002, 37(4):364–375. . 12937557 PMC164367

[3] Freeman MA, Dean MR, Hanham IW. The etiology and prevention of functional instability of the foot. The Journal of bone and joint surgery British 1965, 47(4):678–685. . 5846767

[4] Tropp H, Odenrick P, Gillquist J. Stabilometry recordings in functional and mechanical instability of the ankle joint. Int J Sports Med 1985, 6(3):180–182. doi: 10.1055/s-2008-1025836 . 4030196

[5] Tropp H. Pronator muscle weakness in functional instability of the ankle joint. Int J Sports Med 1986, 7(5):291–294. doi: 10.1055/s-2008-1025777 . 3793339

[6] Willems T, Witvrouw E, Verstuyft J, Vaes P, De Clercq D. Proprioception and Muscle Strength in Subjects With a History of Ankle Sprains and Chronic Instability. J Athl Train 2002, 37(4):487–493. . 12937572 PMC164382

[7] Santos MJ, Liu W. Possible factors related to functional ankle instability. J Orthop Sports Phys Ther 2008, 38(3):150–157. doi: 10.2519/jospt.2008.2524 . 18383650

[8] Cho BK, Park JK, Choi SM, Kang SW, SooHoo NF. The peroneal strength deficits in patients with chronic ankle instability compared to ankle sprain co pers and normal individuals. Foot Ankle Surg 2019, 25(2):231–236. doi: 10.1016/j.fas.2017.10.017 . 29409189

[9] Lim EC, Tan MH. Side-to-side difference in joint position sense and kinesthesia in unilateral functional ankle instability. Foot Ankle Int 2009, 30(10):1011–1017. doi: 10.3113/FAI.2009.1011 . 19796597

[10] Steib S, Pfeifer K. [Sensorimotor Deficits in Functional Ankle Instability]. *Z Orthop Unfall* 2015, 153(3):253–258. doi: 10.1055/s-0034-1396293 . 26008756

[11] Sousa ASP, Leite J, Costa B, Santos R. Bilateral Proprioceptive Evaluation in Individuals With Unilateral Chronic Ankle Instability. J Athl Train 2017, 52(4):360–367. doi: 10.4085/1062-6050-52.2.08 . 28318316 PMC5402534

[12] Docherty CL, Valovich McLeod TC, Shultz SJ. Postural control deficits in participants with functional ankle instability as measured by the balanc e error scoring system. Clin J Sport Med 2006, 16(3):203–208. doi: 10.1097/00042752-200605000-00003 . 16778539

[13] Hiller CE, Refshauge KM, Herbert RD, Kilbreath SL. Balance and recovery from a perturbation are impaired in people with functional ankle instability. Clin J Sport Med 2007, 17(4):269–275. doi: 10.1097/JSM.0b013e3180f60b12 . 17620780

[14] Groters S, Groen BE, van Cingel R, Duysens J. Double-leg stance and dynamic balance in individuals with functional ankle instability. Gait Posture 2013, 38(4):968–973. doi: 10.1016/j.gaitpost.2013.05.005 . 23810093

[15] Laessoe U, Svendsen AW, Christensen MN, Rasmussen JR, Gaml AS. Evaluation of functional ankle instability assessed by an instrumented wobble board. Phys Ther Sport 2019, 35:133–138. doi: 10.1016/j.ptsp.2018.12.002 . 30554122

[16] Vaes P, Van Gheluwe B, Duquet W. Control of acceleration during sudden ankle supination in people with unstable ankles. J Orthop Sports Phys Ther 2001, 31(12):741–752. doi: 10.2519/jospt.2001.31.12.741 . 11767249

[17] Donahue MS, Docherty CL, Riley ZA. Decreased fibularis reflex response during inversion perturbations in FAI subjects. J Electromyogr Kinesiol 2014, 24(1):84–89. doi: 10.1016/j.jelekin.2013.08.012 . 24295544

[18] Sierra-Guzmán R, Jiménez F, Abián-Vicén J. Predictors of chronic ankle instability: Analysis of peroneal reaction time, dynamic balance and isok inetic strength. Clin Biomech (Bristol, Avon) 2018, 54:28–33. doi: 10.1016/j.clinbiomech.2018.03.001 . 29544201

[19] Munn J, Beard DJ, Refshauge KM, Lee RY. Eccentric muscle strength in functional ankle instability. Med Sci Sports Exerc 2003, 35(2):245–250. doi: 10.1249/01.MSS.0000048724.74659.9F . 12569212

[20] Fox J, Docherty CL, Schrader J, Applegate T. Eccentric plantar-flexor torque deficits in participants with functional ankle instability. J Athl Train 2008, 43(1):51–54. doi: 10.4085/1062-6050-43.1.51 . 18335013 PMC2231398

[21] Uh BS, Beynnon BD, Helie BV, Alosa DM, Renstrom PA. The benefit of a single-leg strength training program for the muscles around the untrained ankle. Am J Sports Med 2000, 28(4):568–573. doi: 10.1177/03635465000280042101 . 10921652

[22] Kaminski TW, Buckley BD, Powers ME, Hubbard TJ, Ortiz C. Effect of strength and proprioception training on eversion to inversion strength ratios in subjects w ith unilateral functional ankle instability. Br J Sports Med 2003, 37(5):410–415. doi: 10.1136/bjsm.37.5.410 . 14514531 PMC1751367

[23] Sekir U, Yildiz Y, Hazneci B, Ors F, Aydin T. Effect of isokinetic training on strength, functionality and proprioception in athletes with function al ankle instability. Knee Surg Sports Traumatol Arthrosc 2007, 15(5):654–664. doi: 10.1007/s00167-006-0108-8 . 16770637

[24] Smith BI, Docherty CL, Simon J, Klossner J, Schrader J. Ankle strength and force sense after a progressive, 6-week strength-training program in people with f unctional ankle instability. J Athl Train 2012, 47(3):282–288. doi: 10.4085/1062-6050-47.3.06 . 22892409 PMC3392158

[25] Gribble PA, Delahunt E, Bleakley C, Caulfield B, Docherty CL, Fourchet F, et al. Selection criteria for patients with chronic ankle instability in controlled research: a position sta tement of the International Ankle Consortium. J Orthop Sports Phys Ther 2013, 43(8):585–591. doi: 10.2519/jospt.2013.0303 . 23902805

[26] Sesma AR, Mattacola CG, Uhl TL, Nitz AJ, McKeon PO. Effect of foot orthotics on single- and double-limb dynamic balance tasks in patients with chronic ankle instability. Foot Ankle Spec 2008, 1(6):330–337. doi: 10.1177/1938640008327516 . 19825736

[27] David P, Halimi M, Mora I, Doutrellot PL, Petitjean M. Isokinetic testing of evertor and invertor muscles in patients with chronic ankle instability. J Appl Biomech 2013, 29(6):696–704. doi: 10.1123/jab.29.6.696 . 23343782

[28] Gribble PA, Hertel J. Considerations for Normalizing Measures of the Star Excursion Balance Test %J Measurement in Physical Education and Exercise Science. 2003, 7(2). doi: 10.1207/S15327841MPEE0702_3

[29] Hertel J, Braham RA, Hale SA, Olmsted-Kramer LC. Simplifying the star excursion balance test: analyses of subjects with and without chronic ankle inst ability. J Orthop Sports Phys Ther 2006, 36(3):131–137. doi: 10.2519/jospt.2006.36.3.131 . 16596889

[30] Hyong IH, Kim JH. Test of intrarater and interrater reliability for the star excursion balance test. J Phys Ther Sci 2014, 26(8):1139–1141. doi: 10.1589/jpts.26.1139 . 25202168 PMC4155207

[31] Hiller CE, Refshauge KM, Bundy AC, Herbert RD, Kilbreath SL. The Cumberland ankle instability tool: a report of validity and reliability testing. Arch Phys Med Rehabil 2006, 87(9):1235–1241. doi: 10.1016/j.apmr.2006.05.022 . 16935061

[32] Docherty CL, Moore JH, Arnold BL. Effects of strength training on strength development and joint position sense in functionally unstable ankles. J Athl Train 1998, 33(4):310–314. . 16558526 PMC1320579

[33] Son SM, Kang KW, Lee NK, Nam SH, Kwon JW, Kim K. Influence of Isokinetic Strength Training of Unilateral Ankle on Ipsilateral One-legged Standing Bala nce of Adults. J Phys Ther Sci 2013, 25(10):1313–1315. doi: 10.1589/jpts.25.1313 . 24259783 PMC3820187

[34] Guex K, Daucourt C, Borloz S. Validity and reliability of maximal-strength assessment of knee flexors and extensors using elasticbands. J Sport Rehabil 2015, 24(2):151–155. doi: 10.1123/jsr.2013-0131 . 24700494

[35] Ha SM, Kwon OY, Yi CH, Cynn HS, Weon JH, Kim TH. Effects of scapular upward rotation exercises on alignment of scapula and clavicle and strength of scapular upward rotators in subjects with scapular downward rotation syndrome. J Electromyogr Kinesiol 2016, 26:130–136. doi: 10.1016/j.jelekin.2015.12.007 . 26763601

[36] Hall EA, Docherty CL, Simon J, Kingma JJ, Klossner JC. Strength-training protocols to improve deficits in participants with chronic ankle instability: a ran domized controlled trial. J Athl Train 2015, 50(1):36–44. doi: 10.4085/1062-6050-49.3.71 . 25365134 PMC4299733

[37] Powers ME, Buckley BD, Kaminski TW, Hubbard TJ, Ortiz C. Six Weeks of Strength and Proprioception Training Does Not Affect Muscle Fatigue and Static Balance in Functional Ankle Instability. Journal of Sport Rehabilitation. 2004, 13(3). doi: 10.1123/jsr.13.3.201

