# Supplementary material for: A randomized controlled trial comparing rehabilitation with isokinetic exercises and Thera-Band strength training in patients with functional ankle instability
Source: PLoS One. 2022 Dec 1;17(12):e0278284. doi: 10.1371/journal.pone.0278284 (PMC9714719; doi:10.1371/journal.pone.0278284)
Supplement: S1 File — (DOC) [file pone.0278284.s001.doc]

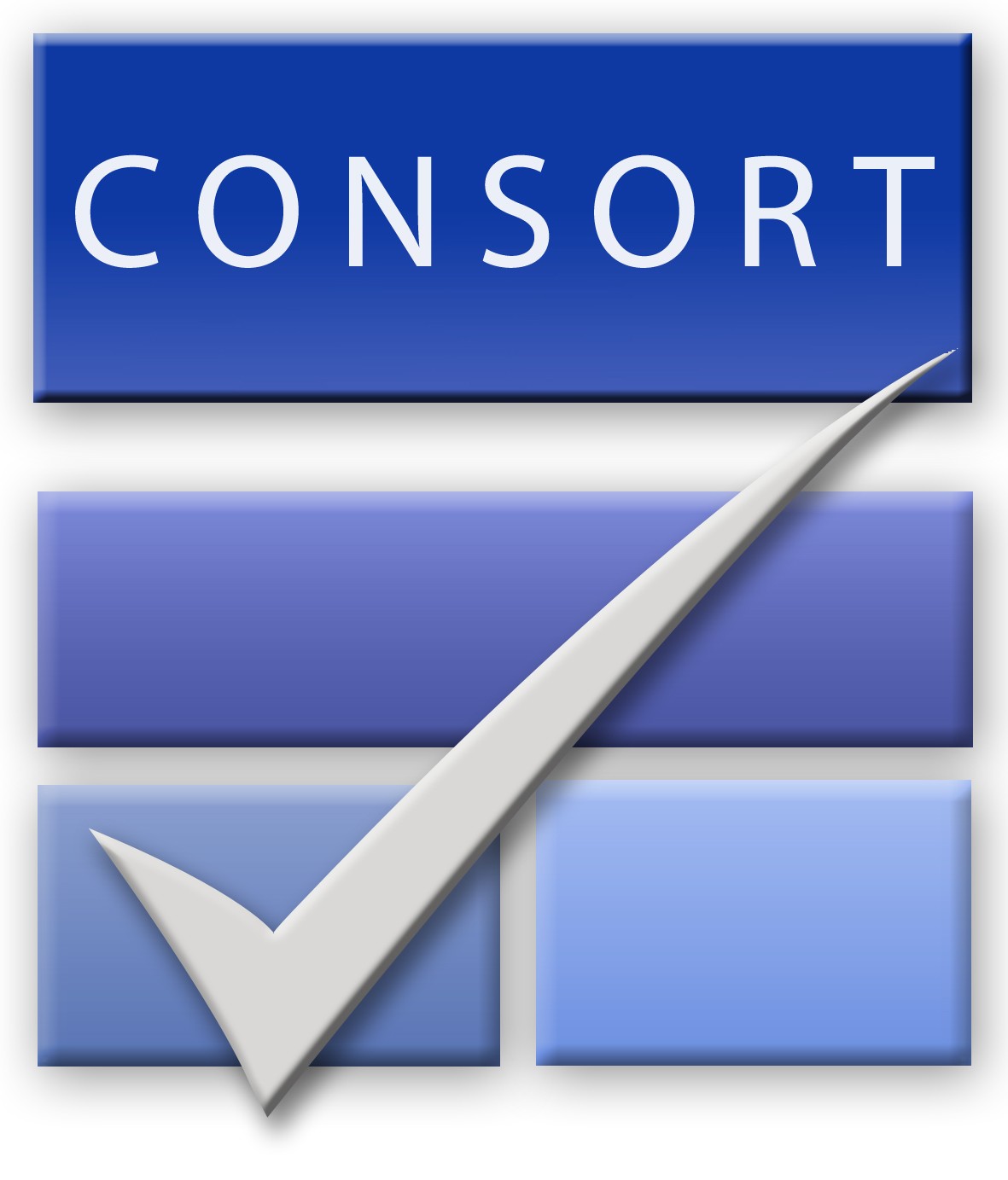
CONSORT 2010 checklist of information to include when reporting a randomised trial*

| Section/Topic | Item No | Checklist item | Reported on page No |
| --- | --- | --- | --- |
| Title and abstract | | | |
|  | 1a | Identification as a randomised trial in the title | Yes Page 1 |
|  | 1b | Structured summary of trial design, methods, results, and conclusions (for specific guidance see CONSORT for abstracts) | Yes Page 2 |
| Introduction | | | |
| Background and objectives | 2a | Scientific background and explanation of rationale | Yes Page 3-4 |
|  | 2b | Specific objectives or hypotheses | Yes Page 4 |
| Methods | | | |
| Trial design | 3a | Description of trial design (such as parallel, factorial) including allocation ratio | Yes Page 5 |
|  | 3b | Important changes to methods after trial commencement (such as eligibility criteria), with reasons | not applicable |
| Participants | 4a | Eligibility criteria for participants | Yes Page 6 |
|  | 4b | Settings and locations where the data were collected | Yes Page 5 |
| Interventions | 5 | The interventions for each group with sufficient details to allow replication, including how and when they were actually administered | Yes Page 9-10 |
| Outcomes | 6a | Completely defined pre-specified primary and secondary outcome measures, including how and when they were assessed | Yes Page 7-9 |
|  | 6b | Any changes to trial outcomes after the trial commenced, with reasons | not applicable |
| Sample size | 7a | How sample size was determined | Yes Page 10 |
|  | 7b | When applicable, explanation of any interim analyses and stopping guidelines | not applicable |
| Randomisation: |  |  |  |
| Sequence generation | 8a | Method used to generate the random allocation sequence | Yes Page 5 |
|  | 8b | Type of randomisation; details of any restriction (such as blocking and block size) | Yes Page 5 |
| Allocation concealment mechanism | 9 | Mechanism used to implement the random allocation sequence (such as sequentially numbered containers), describing any steps taken to conceal the sequence until interventions were assigned | Yes Page 5 |
| Implementation | 10 | Who generated the random allocation sequence, who enrolled participants, and who assigned participants to interventions | Yes Page 5 |
| Blinding | 11a | If done, who was blinded after assignment to interventions (for example, participants, care providers, those assessing outcomes) and how | Yes Page 5 |
|  | 11b | If relevant, description of the similarity of interventions | not applicable |
| Statistical methods | 12a | Statistical methods used to compare groups for primary and secondary outcomes | Yes Page 10 |
|  | 12b | Methods for additional analyses, such as subgroup analyses and adjusted analyses | not applicable |
| Results | | | |
| Participant flow (a diagram is strongly recommended) | 13a | For each group, the numbers of participants who were randomly assigned, received intended treatment, and were analysed for the primary outcome | Yes Page 5 |
|  | 13b | For each group, losses and exclusions after randomisation, together with reasons | not applicable |
| Recruitment | 14a | Dates defining the periods of recruitment and follow-up | Yes Page 10 |
|  | 14b | Why the trial ended or was stopped | not applicable |
| Baseline data | 15 | A table showing baseline demographic and clinical characteristics for each group | Yes Page 11 |
| Numbers analysed | 16 | For each group, number of participants (denominator) included in each analysis and whether the analysis was by original assigned groups | Yes Page 5 |
| Outcomes and estimation | 17a | For each primary and secondary outcome, results for each group, and the estimated effect size and its precision (such as 95% confidence interval) | Yes Page 10 |
|  | 17b | For binary outcomes, presentation of both absolute and relative effect sizes is recommended | not applicable |
| Ancillary analyses | 18 | Results of any other analyses performed, including subgroup analyses and adjusted analyses, distinguishing pre-specified from exploratory | not applicable |
| Harms | 19 | All important harms or unintended effects in each group (for specific guidance see CONSORT for harms) | not applicable |
| Discussion | | | |
| Limitations | 20 | Trial limitations, addressing sources of potential bias, imprecision, and, if relevant, multiplicity of analyses | Yes Page 16 |
| Generalisability | 21 | Generalisability (external validity, applicability) of the trial findings | Yes Page 16 |
| Interpretation | 22 | Interpretation consistent with results, balancing benefits and harms, and considering other relevant evidence | Yes Page 12-15 |
| Other information | | |  |
| Registration | 23 | Registration number and name of trial registry | Yes Page 3 |
| Protocol | 24 | Where the full trial protocol can be accessed, if available | Yes Supporting information |
| Funding | 25 | Sources of funding and other support (such as supply of drugs), role of funders | Yes Supporting information |

*We strongly recommend reading this statement in conjunction with the CONSORT 2010 Explanation and Elaboration for important clarifications on all the items. If relevant, we also recommend reading CONSORT extensions for cluster randomised trials, non-inferiority and equivalence trials, non-pharmacological treatments, herbal interventions, and pragmatic trials. Additional extensions are forthcoming: for those and for up to date references relevant to this checklist, see [www.consort-statement.org](http://www.consort-statement.org/).
