# Supplementary material for: A randomized controlled trial comparing rehabilitation with isokinetic exercises and Thera-Band strength training in patients with functional ankle instability
Source: PLoS One. 2022 Dec 1;17(12):e0278284. doi: 10.1371/journal.pone.0278284 (PMC9714719; doi:10.1371/journal.pone.0278284)
Supplement: S2 File — (DOCX) [file pone.0278284.s002.docx]

**Name of Research Project:** Isokinetic strength training and progressive resistance training with Thera-Band in patients with functional ankle instability

**Scientific Name of The Study:** Which strengthening treatment is more effective for functional ankle instability: isokinetic exercises or Thera-Band strength training?

**Clinical Trial Protocol**

**1.Purpose of research**

The aim of this study was to observe and analyze the differences in ankle strength, dynamic balance ability, and functional improvement in people with functional ankle instability through isokinetic centripetal training and Thera Band strength training for 6 weeks in order to provide new therapeutic ideas for functional rehabilitation of patients with functional ankle instability.

**2. Inclusion and Exclusion Criteria**

The inclusion criteria were as follows:

(1) Patients over the age of 18 years;

(2) Unilateral ankle sprain, anterior drawer and talar tilt test by the same clinician did not find significant structural instability;

(3) Unilateral ankle joint had at least one significant history of lateral ankle sprain in the past 1 year, swelling or pain after injury resulting in the inability of this foot to bear weight normally, and loss of control of the ankle joint during functional activities;

(4) Have an acute ankle sprain that occurred more than 3 months before the enrollment;

(5) There were no other severe injuries in the lower limbs and ankle joints, such as the fractures, surgery, etc.;

(6) The Cumberland Ankle Instability Tool (CAIT) score was < 24 points;

(7) The injured ankle had not received rehabilitation treatments;

(8) Voluntarily participated in this study and signed an informed consent form. Exclusion criteria:

(1) Bilateral ankle sprains;

(2) The fracture or history of surgery in lower limbs;

(3) Ankle joint talar tilt test and anterior drawer test positive, excluding mechanical ankle instability;

(4) Suffering from other neurological diseases affecting muscle strength and balance.

**3. Specific Implementation Steps:**

Control group: training was performed using the progressive resistance protocol of Thera-Band elastic band, and the subjects sat on the floor to receive progressive resistance concentric contraction of ankle dorsiflexors, plantar flexors, invertor and evertor muscles, and only training was replaced with muscle strength around the ankle joint, without compensation of the knee and hip joints. The 170% rest length was chosen as the starting point for the resistance of the Thera-Band elastic band to ensure resistance and standardization (the protocol was designed to ensure that all subjects received consistent strength training).

Experimental group: A multi-joint isokinetic muscle strength test and training system (Guangzhou Yikang Medical Equipment Industrial Co., Ltd. A8-2) device was used. Before training, the subjects remained supine on the seat. According to their height, body size, etc., the equipment was adjusted and fixed in strict accordance with the equipment safety manual. The seat height and power head scale were adjusted according to the parameters provided by the software. The foot movement plane coincided with the foot pedal movement plane. The lateral malleolus remained in a straight line with the power head rotation center. The subject's thigh and foot were fixed on the accessories using nylon rope. First, at an angular velocity of 60 °/s, all subjects were allowed to perform three maximal ankle dorsiflexion and plantar flexion concentric exercises with isokinetic muscle strength testing after 30 min rest to avoid the effects of learning and fatigue. First, dorsiflexors, and plantar flexors muscle strength training was performed, and ankle inversion and eversion isokinetic muscle strength training was performed after 5 minutes of rest. The training procedure was performed according to the A8-2 training manual for concentric/concentric contractions with an angular velocity of 60 °/s. Subjects exercised ankle dorsiflexion and plantar flexion muscle strength in the dorsiflexion/plantar flexors movement mode and ankle inversion and eversion muscle strength in the inversion/eversion movement mode.

Both the experimental group and the control group were trained three times a week for a total of 6 weeks.

**4. Judgment and Evaluation Methods of Experimental Research Efficacy**

Before the training, all subjects underwent the CAIT score, isokinetic muscle strength and dynamic balance test. The IST group and TBT group all received prescribed muscle strength exercise programs of 3d/week for 6 weeks. All the tests and exercise were completed in the Sports Medicine Department of the Affiliated Xuzhou Rehabilitation Hospital of Xuzhou Medical University. After six weeks, all subjects were reassessed using the following outcome measures: isokinetic muscle strength, dynamic balance.

**(1) Strength test**

A multi-joint isokinetic muscle strength testing and training system (Guangzhou Yikang Medical Equipment Industrial Co., Ltd. A8-2 type, China) device was used in this study. Before the test, subjects were kept in a supine position on the seat. According to the height, body size, etc., the equipment was adjusted and fixed in accordance with the equipment safety manual strictly. First, under the condition of 60 °/s angular velocity, all subjects were allowed to perform three maximal concentric exercises of ankle dorsiflexion and plantarflexion. Then subjects were randomly divided into IST group and TBT group for the isokinetic muscle strength test after 30 minutes of rest to avoid the effects of learning and fatigue. Under the conditions of 60 °/s and 120 °/s angular velocity, the subjects respectively completed 10 consecutive repeated maximum isokinetic concentric contractions of ankle dorsiflexion and plantarflexion, inversion and eversion, and the peak torque and peak torque/body weight were recorded. Selection of evaluation indicators were as follows: relative peak torque (RPT), ratio of peak torque to individual body mass. In this study, we eliminated the effect of individual body mass on muscle strength, which can be better used to compare the differences in muscle strength caused by weight differences.

**(2) Balance test**

The Star Excursion Balance Test (SEBT) was used for balance test, which had sufficient sensitivity and high retest reliability in ankle dynamic balance test. The specific test methods were as follows: firstly, leg length was measured from the anterior superior iliac spine to the medial malleolus on physical examination by the same therapist; second, subjects were asked to stand barefoot with the navicular of their stance limb positioned over the center of the SEBT tape grid, placing both hands on the waist and bearing weight on the unilateral lower limb to maintain physical stability. At the same time, the other lower limb extended to the limit in eight directions: anterior, anteromedial, medial, posteromedial, posterior, posterolateral, lateral, anterolateral. The maximal distance that the foot was lightly touched in each direction was recorded, and more distance meant more stable in ankle joint. The ratio of maximal extension distance to leg length was calculated as an index of dynamic balance capability.

**(3) CAIT Scoring**

Measures of FAI were obtained using the Cumberland Ankle Instability Tool (CAIT), consisting of nine questions with high reliability and validity. Before the trial, the CAIT was used to assess the severity of FAI in subjects, and rated them again according to their recovery status after the end of training.  Before distributing the questionnaires, the researchers introduced themselves and explained the plan and the questions to the subjects.

**5. Statistical Analysis**

**(1)** Statistical analysis was performed using IBM SPSS Statistics 25 software (SPSS Inc, Chicago, Illinois). The level of significance was set at P < 0.05. The normality of each variable was initially tested with the Kolmogorov-Smirnov test. The chi-square test was used for gender comparison. The independent sample t-test was used for the comparison between two groups, while the paired t-test for the comparison within the group.

**(2)** sample size

The sample size was estimated with the G*Power 3.1.9.2 software based on some similar studies. Consequently, using a power with 80% beta = 0.2, alpha = 0.05 and an estimated effect size = 0.7, the estimated sample size of 26 subjects were in per group (total = 52).

**6. Technology Roadmap**

**
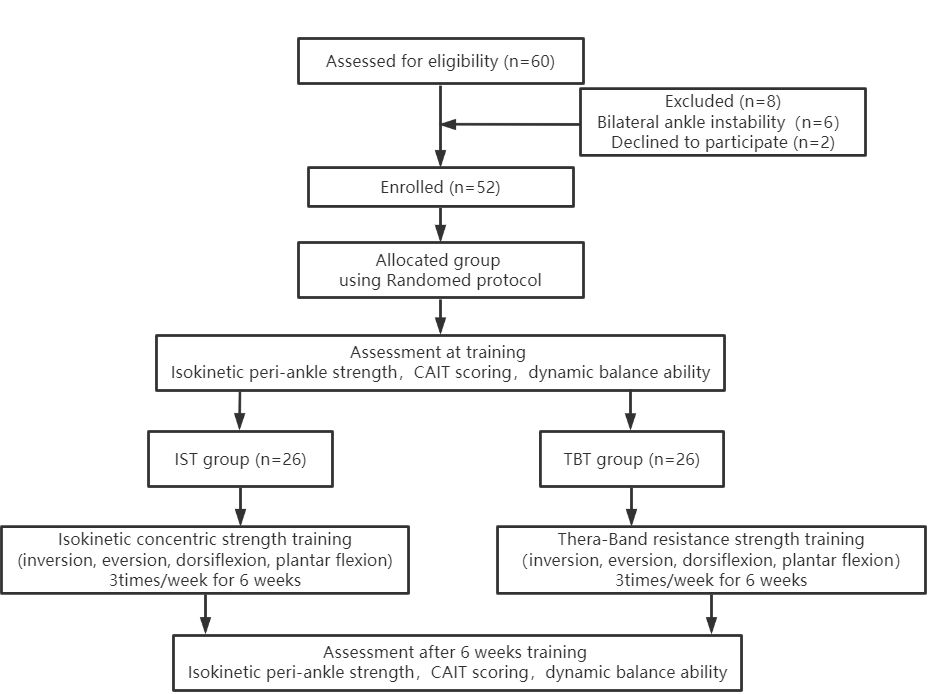
**
