# Supplementary material for: A randomized controlled trial comparing rehabilitation with isokinetic exercises and Thera-Band strength training in patients with functional ankle instability
Source: PLoS One. 2022 Dec 1;17(12):e0278284. doi: 10.1371/journal.pone.0278284 (PMC9714719; doi:10.1371/journal.pone.0278284)
Supplement: S3 File — (PDF) [file pone.0278284.s003.pdf]

项目名称：等速肌力训练在改善功能性踝关节不稳的应用研究

研究方案名称：功能性踝关节不稳患者的等速力量训练和 Thera-Band 渐进阻力训练

临床试验方案

（一）研究目的：本研究旨在通过等速向心力量训练和 Thera Band 力量训练 6 周，观察和分析功能性踝关节不稳人群在踝关节力量、动平衡能力和功能改善方面的差异，以期为功能性踝关节不稳患者功能康复提供新的治疗思路。

（二）研究对象纳入标准：

纳入标准如下：

(1)18 岁以上患者；

(2)同一位临床医生进行的单侧踝关节扭伤、前抽屉和距骨倾斜试验未发现明显的结构不稳定；

(3)单侧踝关节在过去 1 年中至少有一次明显的踝关节外侧扭伤史，受伤后肿胀或疼痛导致该足无法正常负重，以及在功能活动期间踝关节失去控制；

(4)上一次踝关节扭伤发生在参与实验前 3 个月；

(5)下肢及踝关节无骨折、手术等其他严重损伤。

(6) 坎伯兰踝关节不稳工具（CAIT）评分<24 分；

(7)受伤脚踝未接受康复治疗；

(8)自愿参与本研究并签署知情同意书。

排除标准：

(1)双侧踝关节扭伤；

(2)下肢骨折或手术史；

(3)踝关节距骨倾斜试验和前抽屉试验阳性，不包括机械性踝关节不稳；

(4)患有影响肌肉力量和平衡的其他神经疾病。

（三）具体实施步骤：

对照组：使用 Thera-Band 弹力带的渐进阻力方案进行训练，受试者坐在地板上接受踝关节背屈肌、跖屈肌、内翻肌和外翻肌的渐进阻力向心收缩，并且只训练换成踝关节周围肌力，而不进行膝关节和髋关节的代偿。选择 170%的静止长度作为 Thera-Band 弹力带阻力的起点，以确保更大的阻力和标准化（该方案的设计是为了确保所有受试者都接受一致的力量训练）。

实验组：采用多关节等速肌肉力量测试与训练系统(广州一康医疗设备实业有限公司 A8-2 型) 装置，训练前，受试者在座椅上保持仰卧位，根据其身高、体型等，严格按照设备安全使用手册调整并固定设备，按照软件所提供的参数进行座椅高度和动力头刻度调节，将足移动平面和足部踏板移动平面重合，外踝与动力头转动中心保持在一条直线上，使用尼龙绳将受试者大腿和足固定在配件上。首先，在 60°/s 角速度的条件下，所有受试者被允许进行三次最大的踝关节背屈和跖屈同心运动，在休息 30 分钟后进行等速肌力测试，以避免学习和疲劳的影响。首先进行背屈、跖屈肌力训练，休息 5 分钟后进行踝关节内翻、外翻等速肌力训练。训练程序按照 A8-2 训练手册进行向心/向心收缩，角速度为 60°/s。受试者在背屈/跖屈运动模式下锻炼踝关节背屈和跖屈肌肉力量，在内翻/外翻运动模式下锻炼踝关节内翻和外翻肌肉力量。

试验组和对照组都进行每周三次，共持续 6 周的训练方案。

（四）实验研究疗效判断及评估方法：

训练前，所有受试者均接受 CAIT 评分、等速肌力和动态平衡测试。对照组和实验组均接受规定的 3d/周肌力锻炼计划，持续 6 周。所有测试和锻炼均在徐州医科大学附属徐州康复医院运动医学科完成。六周后，所有受试者使用以下结果指标重新评估：等速肌力、动态平衡、踝关节功能。

1.肌力评估

测试前，受试者在座椅上保持仰卧姿势。根据高度、体型等，严格按照设备安全手册进行调整和固定。首先，在 60°/s 角速度的条件下，所有受试者被允许进行三次最大的踝关节背屈和跖屈同心运动。在休息 30 分钟后进行等速肌力测试，以避免学习和疲劳的影响。在 60°/s 和 120°/s 角速度条件下，受试者分别完成 10 次连续的踝关节背屈和跖屈、内翻和外翻的最大等速向心收缩，并记录峰力矩和峰力矩/体重。评价指标的选择如下：相对峰值扭矩（RPT）、峰值扭矩与个体体重之比。在这项研究中，我们消除了个体体重对肌力的影响，这可以更好地用于比较体重差异引起的肌力差异。

2. 平衡测试采用星漂移平衡测试(Star Excursion Balance Test, SEBT)，在踝关节动态平衡测试中具有足够的灵敏度和较高的重测信度。具体测试方法如下：首先，由同一治疗师在体检时测量从髂前上棘到内踝的腿长；第二，要求受试者赤脚站立，站立肢体的舟状骨位于 SEBT 胶带网格的中心上方，双手放在腰部，单侧下肢负重，以保持身体稳定性。同时，另一条下肢向八个方向伸展至极限：

前、前内侧、内侧、后内侧、后、后外侧、外侧、前外侧。记录了脚在各个方向轻轻触碰的最大距离，距离越远，踝关节越稳定。计算最大伸展距离与腿长之比，作为动态平衡能力的指标。

3. 功能性踝关节不稳的个体功能是使用坎伯兰踝关节不稳工具（Cumberland Ankle Instability Tool，CAIT）获得的，该工具由九个问题组成，具有较高的信度和效度。试验前，CAIT 用于评估受试者 FAI 的严重程度，并根据训练结束后的恢复状态再次对其进行评级。在分发问卷之前，研究人员自我介绍，并向受试者解释了计划和问题。

（五）统计学分析：

1.使用 IBM SPSS Statistics 25 软件（伊利诺伊州芝加哥市 SPSS 公司）进行统计分析。显著性水平设定为 $P<0.05$。每个变量的正态性最初通过 Kolmogorov-Smirnov 检验进行检验。性别比较采用卡方检验。两组间比较采用独立样本 t 检验，组内比较采用配对 t 检验。

2.样本量计算

根据一些类似的研究，使用 G*Power 3.1.9.2 软件估计样本量。因此，使用 80% $\beta=0.2$、$\alpha=0.05$ 和估计效应大小=0.7，每组 26 名受试者的估计样本（总数=52）。

（六）技术路线图

严格按照入组标准筛选患者

治疗前用等速力量测试系统评估踝关节肌力，用星偏移平衡测试评估踝关节稳定性，用坎伯兰踝关节不稳工具评估患者踝关节功能.

对照组：Thera-Band 弹力带的渐进阻力方案
3d/周肌力锻炼计划，持续 6 周

实验组：等速肌力训练
3d/周肌力锻炼计划，持续 6 周

受试者均在治疗 6 周后再次进行评估：包含踝关节趾屈、背伸、内翻、外翻肌力，用星偏移平衡测试评估踝关节稳定性，同时评估踝关节各关节活动范围、坎伯兰踝关节不稳工具评估患者踝关节功能。

对各组数据进行处理、分析比较
得出结论
